# Genomic selection signatures in farmed *Colossoma macropomum* from tropical and subtropical regions in South America

**DOI:** 10.1111/eva.13351

**Published:** 2022-02-24

**Authors:** John Fredy Gómez Agudelo, Vito Antonio Mastrochirico‐Filho, Carolina Heloisa de Souza Borges, Raquel Belini Ariede, Lieschen Valeria Guerra Lira, Rubens Ricardo de Oliveira Neto, Milena Vieira de Freitas, Gustavo Adolfo Lenis Sucerquia, Manuel Vera, Milthon Honorio Muñoz Berrocal, Diogo Teruo Hashimoto

**Affiliations:** ^1^ Aquaculture Center of UNESP São Paulo State University (UNESP) Jaboticabal Brazil; ^2^ Facultad de Ciencias Agrarias Universidad de Antioquia Medellín Colômbia; ^3^ Facultad de Veterinaria Universidad de Santiago de Compostela (USC) Lugo Spain; ^4^ 125811 Facultad de Zootecnia Universidad Nacional Agraria de la Selva Tingo Maria Perú

**Keywords:** genetic structure, neotropical fish, serrasalmidae, signatures of selection, South American Aquaculture, stress in aquaculture

## Abstract

Tambaqui or cachama (*Colossoma macropomum*) is one of the most important neotropical freshwater fish used for aquaculture in South America, and its production is concentrated at low latitudes (close to the Equator, 0°), where the water temperature is warm. Therefore, understanding how selection shapes genetic variations and structure in farmed populations is of paramount importance in evolutionary biology. High‐throughput sequencing to generate genome‐wide data for fish species allows for elucidating the genomic basis of adaptation to local or farmed conditions and uncovering genes that control the phenotypes of interest. The present study aimed to detect genomic selection signatures and analyze the genetic variability in farmed populations of tambaqui in South America using single‐nucleotide polymorphism (SNP) markers obtained with double‐digest restriction site‐associated DNA sequencing. Initially, 199 samples of tambaqui farmed populations from different locations (located in Brazil, Colombia, and Peru), a wild population (Amazon River, Brazil), and the base population of a breeding program (Aquaculture Center, CAUNESP, Jaboticabal, SP, Brazil) were genotyped. Observed and expected heterozygosity was 0.231–0.350 and 0.288–0.360, respectively. Significant genetic differentiation was observed using global *F*ST analyses of SNP loci (*F*ST = 0.064, *p* < 0.050). Farmed populations from Colombia and Peru that differentiated from the Brazilian populations formed distinct groups. Several regions, particularly those harboring the genes of significance to aquaculture, were identified to be under positive selection, suggesting local adaptation to stress under different farming conditions and management practices. Studies aimed at improving the knowledge of genomics of tambaqui farmed populations are essential for aquaculture to gain deeper insights into the evolutionary history of these fish and provide resources for the establishment of breeding programs.

## 1 INTRODUCTION

Tambaqui or cachama (*Colossoma macropomum*) is a fish species that primarily inhabits the floodplains of the Amazon and Orinoco basins in South America (Araujo‐Lima & Goulding, 1997). Tambaqui is a tropical fish, which typically prefers warm waters (Woynárovich & Van Anrooy, 2019), such as those in their natural habitats, where the temperature is above 26°C (e.g., Central Amazon; Affonso et al., 2015). Farming of this species began in the 1960s, and currently, it is considered the main native South American fish for aquaculture farmed under diverse environmental conditions. The leading producers include Brazil, Peru, and Colombia (Woynárovich & Van Anrooy, 2019). Production primarily occurs on small or medium fish farms using semi‐intensive and intensive systems. In 2019, tambaqui accounted for nearly 19% (101,100 tons) of total Brazilian fish production (the major producer). Owing to its high rusticity, rapid growth, high productivity, and substantial commercial value in the international markets (IBGE, 2020; Valladão et al., 2018), tambaqui is one of the most important neotropical freshwater fish used for aquaculture in South America. Despite its smaller proportion, tambaqui production in Colombia and Peru has increased over the last few decades (Valladão et al., 2018). Nowadays, tambaqui is also being farmed in Asian countries, particularly China, Indonesia, Malaysia, Myanmar, and Vietnam (Valladão et al., 2018; Woynárovich & Van Anrooy, 2019).

Domestication is considered a prolonged process that allows for the adaptation of new species to captive environments. Selective breeding focusing on economic and adaptive traits may be applied to improve the productivity of farmed fish (Teletchea, 2018; Teletchea & Fontaine, 2014). However, even today, almost all tambaqui production from aquaculture in South America relies upon stocks that are not completely domesticated, and wild populations serve as a source to maintain farmed breeding populations (Fazzi‐Gomes et al., 2017; Santos et al., 2007). Farming conditions, such as high stocking density, climatic fluctuations, and stressful management, negatively affect tambaqui production by suppressing immunity, enhancing disease susceptibility, and increasing mass mortality (Boijink et al., 2016). Apparently, tambaqui individuals exhibit a poorer growth performance in Brazilian subtropical regions (South Brazil), where temperatures are below 23°C. Moreover, fish die if the water temperature remains below 15°C for several days (Zaniboni‐Filho & Meurer, 1997). These temperature limitations impede the natural range expansion and establishment of the species in waters where temperature drops below 15–18°C during cold seasons (Woynárovich & Van Anrooy, 2019). Similarly, abrupt seasonal temperature variations in Brazilian subtropical regions, together with stresses triggered by intensive production, can result in the outbreak of diseases, such as those caused by the ectoparasite *Ichthyophthirius multifiliis* (Lira et al., 2020).

Genomic breeding programs for tambaqui were initiated in the first decade of the 21^st^ century, with emphasis on improving the growth performance and disease resistance of these fish (Ariede et al., 2020; De Mello et al., 2015; Lira et al., 2020; Perazza et al., 2019). Although the assessment of genetic diversity is a fundamental step in the introduction of genomic breeding programs (Mastrochirico‐Filho et al., 2019), there have been few large‐scale population genetic studies of farmed populations (Mastrochirico‐Filho et al., 2021; Nunes et al., 2017, 2020). Furthermore, no study has focus on the efficient breeding management of tambaqui, as opposed to a closely related serrasalmid species, pacu (*Piaractus mesopotamicus*) (Freitas et al., 2021). In addition, majority of the genetic studies aimed at understanding the degree of genetic variability and population structure were restricted to Brazilian stocks. Specifically, studies based on mitochondrial DNA and microsatellites have revealed moderate genetic differentiation among tambaqui broodstocks and loss of genetic variability in captivity, due perhaps to the small size of the founding populations and lack of appropriate breeding management (Aguiar et al., 2018; Ferreira et al., 2019; Gonçalves et al., 2018). As such, the formation of breeding units with unknown genetic variability in farmed populations may lead to the loss of genetic potential and an increase in inbreeding risk (Charlesworth & Willis, 2009; Melo et al., 2006). For instance, in rainbow trout (*Oncorhynchus mykiss*), different degrees of inbreeding depression in stocks decreased hatching, fecundity, and larval survival rates and generated individuals with morphological deformities (Yousefian & Nejati, 2008).

Genomic techniques, such as double‐digest restriction site‐associated DNA sequencing (ddRAD‐Seq), can be applied for the discovery and genotyping of thousands of single nucleotide polymorphisms (SNPs) simultaneously. Moreover, ddRAD‐Seq could be employed in genomic studies of several aquaculture species, even when limited genomic information was available (Houston et al., 2020; Robledo et al., 2018). The discovery and characterization of SNP markers have assumed relevance in genetic studies aimed at investigating the diversity, differentiation, and structure of wild and cultivated stocks for the expansion of aquaculture to several nonmodel South American species (Mastrochirico‐Filho et al., 2019; Torati et al., 2019).

Furthermore, genomic approaches have been applied to detect selection signatures, which may be functionally linked to genetic variations in traits subject to selection (Lopez et al., 2015). In recent years, selection signatures have been intensively studied because of their potential association with genes that control the phenotypes of interest in farmed populations of fish (Gutierrez et al., 2016; López et al., 2019). However, contrary to the production of salmon, which has been intensively selected for economically important traits since the 1970s (Gjedrem, 2010), the domestication of tambaqui is relatively recent and involves farmed populations that have not undergone strong artificial selection as yet. Therefore, considering that the power of detection of SNP outliers depends primarily on the number of generations since domestication and the strength of selection (Gutierrez et al., 2016), novel insights into selection signatures focused on tambaqui production will expand our knowledge of gradual changes at the genomic level in populations adapted to farmed conditions, which may be associated with the traits of commercial interest. Additionally, the detection of novel selection signature may allow for the identification of genes formerly associated with farmed environments, which can serve as resources for increasing the productivity of this native South American fish species (Gutierrez et al., 2016; López et al., 2019; Vera et al., 2019).

To this end, the present study aimed to investigate the genetic diversity and population structure of farmed populations of tambaqui in commercial hatcheries from the leading producers of this species in South America (i.e., Brazil, Colombia, and Peru). Furthermore, the genomic selection signatures were investigated to identify the regions associated with the variations in economic traits and/or adaptation to captive environments in farmed tambaqui in tropical and subtropical regions of South America.

## 2 MATERIALS AND METHODS

### 2.1 Sampling

Samples were obtained from 361 tambaqui breeders across 12 commercial fish farms in Brazil (BR), Colombia (COL), and Peru (PER) as well as a wild population from the Amazon River (3°29′S, 60°66′W) (WILD). A base population used in breeding program research conducted by the Laboratory of Genetics in Aquaculture and Conservation (LaGeAC) at the Universidade Estadual Paulista (UNESP), Jaboticabal (São Paulo State, Brazil) (BRGEN), was also sampled. This population includes individuals from different fish farms and is studied to obtain genomic information, aimed at advancing aquaculture breeding programs for native fish species, with a particular focus on improving growth performance and disease resistance (Table 1).

**TABLE 1:** Parameters of genetic diversity in farmed populations of tambaqui *Colossoma macropomum* considering 1633 SNPs

Sampling site	*N*	N_a_	H_o_ (SD)	H_e_ (SD)	*F*_IS_	HWE	MAF > 0.1 (%)
BR1	14	1.968	0.350 (0.198)	0.351 (0.140)	0.060	1623	1401 (85.8)
BR2	19	1.975	0.314 (0.164)	0.349 (0.138)	0.131	1614	1409 (86.3)
BR3	20	1.966	0.231 (0.139)	0.345 (0.139)	0.357	1527	1408 (86.2)
BR4	11	1.983	0.311 (0.164)	0.358 (0.129)	0.193	1632	1452 (88.9)
BR5	15	1.950	0.256 (0.159)	0.342 (0.145)	0.287	1600	1394 (85.4)
BR6	18	1.971	0.290 (0.154)	0.345 (0.138)	0.190	1616	1.395 (85.4)
BR All	97	2.000	0.290 (0.109)	0.368 (0.112)	0.225	1195	1521 (93.1)
BRGEN	15	1.985	0.306 (0.160)	0.353 (0.131)	0.170	1616	1452 (88.9)
COL1	19	1.944	0.283 (0.179)	0.336 (0.152)	0.190	1592	1338 (81.9)
COL2	14	1.897	0.260 (0.183)	0.321 (0.164)	0.234	1609	1278 (78.3)
COL3	16	1.985	0.293 (0.157)	0.360 (0.130)	0.222	1603	1444 (88.4)
COL4	12	1.878	0.250 (0.183)	0.312 (0.168)	0.247	1617	1239 (75.9)
COL5	6	1.775	0.263 (0.236)	0.288 (0.185)	0.188	1633	1154 (70.6)
COL All	67	1994	0.274 (0.132)	0.351 (0.134)	0.230	1294	1410 (86.3)
PER	14	1.954	0.249 (0.157)	0.343 (0.144)	0.325	1601	1380 (84.5)
WILD	6	1.908	0.263 (0.195)	0.337 (0.152)	0.330	1633	1371 (84.0)

Note

Standard deviation values (SD) are between parentheses.

Abbreviations: F_IS_, inbreeding coefficient; H_e_, expected heterozygosity; H_o_, observed heterozygosity; HWE, loci in Hardy–Weinberg equilibrium after FDR‐BY correction (*p*‐adjusted > 0.0063);MAF, minimum allelic frequency; *N*, sampling size; N_a_, average number of alleles per loci.

The sampled farmed populations cannot be considered fully domesticated, as they are still based on genetically unselected fish. Farmed populations from Brazil (BR1 to BR6) correspond to samples from subtropical regions, which are characterized by wide variations in temperature (maximum temperatures during summer typically exceed 30°C and minimum temperatures during winter are below 15°C). Farmed populations from Colombia (COL1 to COL5) and Peru (PER) correspond to samples from the equatorial regions (tropical) of South America, which are characterized by constant and high mean temperatures (~30°C) with very little annual variations. Unfortunately, environmental variables were not recorded during the collection period of farmed fish.

The fish farms in Brazil and Colombia are over 20 years old and have adopted intensive breeding systems in large facilities, with more intensified management to supply the increasing demand. The fish farms in Peru are relatively recent (10‐year‐old) and considered less developed than those in other main South American producers. Furthermore, Peruvian aquaculture is dominated by marine species, and tambaqui production remains small scale and at the subsistence level. The commercial identity and localization of the fish farms are confidential. The number of samples collected per population is presented in Table 1. The fish were individually tagged with pit tags (passive integrated transponder tags, FDX‐B full‐duplex model, 134.2 kHz) and maintained in a farm for subsequent analyses.

All procedures were performed with strict adherence to the recommendations of the National Council for Control of Animal Experimentation (CONCEA) (Brazilian Ministry for Science, Technology and Innovation), and the study approved by the Animal Use Ethics Committee (CEUA), number 019006/17. Fin samples were collected from each fish under anesthesia administered using benzocaine solution (200 mg·L^−1^) (Sigma, St. Louis, USA), and all efforts were made to minimize suffering. Fin samples were stored in 95% ethanol at −20°C.

### 2.2 ddRAD‐Seq library construction

Prior to genomic analysis, a purity analysis was performed using species‐specific multiplex polymerase chain reaction (PCR) (Hashimoto et al., 2011) to ensure that the samples originated from pure tambaqui specimens and not from interspecific hybrids. This preanalysis was applied because in Colombian and Brazilian hatcheries, hybrid individuals are commonly produced from one or two serrasalmid species, such as pirapitinga (*Piaractus brachypomus*) and pacu (*Piaractus mesopotamicus*).

The ddRAD‐Seq libraries for SNP identification were constructed based on the protocol originally described by Peterson et al. (2012). Briefly, genomic DNA was extracted from 361 individuals using the PureLink Genomic DNA Kit. Each library was constructed using 45 individuals identified with barcodes (sequences of six base pairs). A total of eight libraries were constructed. The amount of genomic DNA was determined using Qubit^®^ 3.0 (Thermo Scientific™) and standardized to the concentration of 10 ng·µL^−1^. The purity of the nucleic acid samples was determined using spectrophotometry with NanoDrop™ One (Thermo Scientific™), and the absorbance ratio (A260/A280) was between 1.80 and 2.00. DNA integrity of the samples was visualized using 1% agarose gel electrophoresis. Genomic analyses of data from COL5 and WILD were restricted to a few individuals and affected by the difficulty in obtaining DNA from biological samples. For DNA digestion, the final reaction mixture contained 2.5 µl of DNA (totaling 25 ng) digested with 0.1 µl (10 U·ml^−1^) of the restriction enzyme MluCI (5′‐…AATT…‐3′) and 0.1 µl (20 U·ml^−1^) of the restriction enzyme SphI (5′‐…GCATGC…‐3′); 0.6 µl of the CutSmart 10× buffer; and 2.8 µl of water. The conditions for enzyme digestion were 37°C for 60 min. Based on the genome of tambaqui (GCA_904425465.1), computer simulations were performed to evaluate the cut frequency for each restriction enzyme using Nebcutter v2.0 (Vincze, 2003). The average distance between enzymatic sites of enzyme was 6500 bp for SphI and 250 bp for MluCI, which allowed for adjusting the amount of specific adapters for each enzyme, calculated as described by Peterson et al. (2012). Ligation was performed in a final volume of 10.0 µl, containing 0.2 µl of the T4 ligase enzyme, 1.0 µl of the 10× T4 DNA ligase reaction buffer, 1.0 µl of the P1 adapter (4.04 µM), and 1.0 µl of the P2 adapter (0.07 µM). For binding, the samples were incubated at 23°C for 90 min, followed by 65°C for 10 min. Short fragments resulting from DNA digestion and adapter ligation were removed using the Agencourt AMPure XP cleanup step Kit (Beckman Coulter, DE), following the manufacturer's protocol. This step allowed us to pool all samples for each library, and the libraries were then used to select specific fragments. Fragments of approximately 450 bp were selected using the E‐Gel^®^ Size Select 2% agarose gel (Invitrogen).

The enrichment step was then performed via PCR amplification using the Phusion^®^ High‐Fidelity PCR Kit. This step was necessary to incorporate the Illumina flow cell sequence and regions of the Illumina primer sequences. To increase the number of DNA fragments, 20 independent PCR assays were performed. All reactions were performed in a final volume of 20.0 µl, containing 9.5 µl of H_2_O, 0.4 µl of 10‐mM dNTPs, 4.0 µl of 5× Phusion HF Buffer, 2.5 µl of P1 5.0 µM Primer, 2.5 µl of P2 5.0 µM Primer, 0.12 µl of Phusion DNA Polymerase, and 1.0 µl of digested DNA. All reactions involved an initial stage of 68°C for 1 min, followed by 12 cycles at 95°C for 10 s, 60°C for 30 s, and 72°C for 30 s, and a final stage of 72°C for 7 min. The PCR samples were grouped in a final volume of 400 µl and were repurified using AMPure XP beads (Beckman Coulter, DE), according to the manufacturer's protocol. The pool of amplified sequences was analyzed by agarose gel electrophoresis. The concentration of each final library was verified by fluorometry using Qubit^®^ 3.0 (Thermo Scientific™). The samples were sequenced on the Hiseq 4000 platform, generating 1.7 G (billions) of paired‐end reads.

### 2.3 SNP identification and filtering

Stacks v. 2.1 (Catchen et al., 2011, 2013) was used to analyze the raw sequences resulting from Illumina sequencing. Following the Stacks manual (https://catchenlab.life.illinois.edu/stacks/manual/#align), the analysis was initiated with the “process_radtags” module, in which the barcodes were assigned to individuals, and low‐quality sequences and adapters were excluded. After demultiplexing and cleaning, the data were aligned against the tambaqui reference genome (GCF_904425465.1) using Burrows–Wheeler Aligner (BWA, v0.7.15) with the standard parameters (Li & Durbin, 2009). The mapped regions were used to identify genomic variants using Samtools (Li et al., 2009) and Vcftools (Petr et al., 2011), which filtered them based on a general quality score of >20 and minimum allele count of ≥4. Samples with over 40% missing genotypes were removed (mind 0.4) using Plink v. 1.07 (Purcell et al., 2007). Quality control filters were also applied using Plink v. 1.07 (Purcell et al., 2007), excluding SNPs that did not pass in the following criteria: missing genotypes (geno) < 0.3 and minor allele frequency (MAF) < 0.05. Furthermore, the possible genotyping errors were discarded using Vcftools (Petr et al., 2011), removing SNPs deviating from the Hardy–Weinberg equilibrium considering HWE *p*‐value < 1 × 10^−6^. Finally, the filtered SNPs were present in at least 70% individuals in a population (call rate > 0.70). The resulting variants were filtered to consider only one SNP per locus.

### 2.4 Genetic diversity analyses

The genetic diversity parameters were estimated considering the geographic regions (Brazil, Colombia, and Peru) and sampled fish farms. All remaining SNPs were pruned for linkage disequilibrium (LD) using the indep‐pairwise option (‐‐indep‐pairwise 50 10 0.1) in Plink v. 1.07 (Purcell et al., 2007) to avoid the effects of linked SNPs. The observed (H_o_) and expected (H_e_) heterozygosity, average number of alleles per locus (N_a_), and MAF were estimated using Plink v. 1.07 (Purcell et al., 2007). To assess heterozygote deficiency in a fish farm, deviations from the Hardy–Weinberg equilibrium (HWE *p*‐value < 0.05) were predicted with the exact test in Plink v. 1.07 (Purcell et al., 2007), using the FDR‐BY correction method described to Narum (2006). The inbreeding coefficient (*F*_IS_) for each fish farm was estimated using the R package hierfstat (Goudet & Jombart, 2015). The contemporary effective population size (N_e_) for each fish farm was calculated using the linkage disequilibrium method in NeEstimator V2.01 (*p*‐value < 0.05) (Do et al., 2014). For this analysis, rare alleles were avoided while retaining SNPs with the highest polymorphic content (PIC) and MAF > 0.1 (Marandel et al., 2020). Identity by state (IBS) distances by multidimensional scaling (MDS) analysis were performed using Plink v. 1.07 (Purcell et al., 2007) to estimate the genomic relatedness and similarity between individuals from different geographic regions.

The genetic structure of fish farms from different countries was determined based on the global and pairwise estimates of *F*ST using hierfstat (Goudet & Jombart, 2015) and Arlequin 3.5 (Excoffier & Lischer, 2010). The genetic distances between stocks were measured as described by Nei (1972). Mixing levels between stocks were inferred by estimating the most probable number of population units (K) using Structure V2.3.4 (Falush et al., 2007), without prior information of the population. First, the distribution of ∆K was determined, and an ad hoc statistic based on the rate of change in the logarithmic probability of data between successive K values was used. The cluster interval (K) was predefined from 1 to 14. The analysis was performed with 70 replicates (i.e., five repetitions for each K value) and 50,000 iterations after 100,000 burn‐in steps. The most probable value of K to explain the population structure was the modal value of ∆K, as described by Evanno et al. (2005). The results of structure analysis were viewed using Structure Harvester (Earl & Von Holdt, 2012). Discriminant analysis of principal components (DAPCs) (Jombart et al., 2010) was also used to examine the population structure with the R package adegenet (Jombart, 2008), describing the genetic clusters considering the geographic location of the fish farms as predefined groups. The find.clusters function was applied to determine the number of clusters, and the xvalDAPC and optim.a.score functions were used to calculate the optimal number of principal components to be retained for the analysis. Analysis of molecular variance (AMOVA) was performed in Arlequin 3.5 (Excoffier & Lischer, 2010) (10,000 permutations to test significance) considering the clusters obtained in structure analysis and DAPC.

### 2.5 Identification of SNP outliers

Three methods were used for the identification of SNP outliers in the populations, considering groups compounding all commercial fish farms from Brazil (group BR), Colombia (group COL), and Peru (group PER). BRGEN and WILD were excluded from the analysis. Subsequently, data from the adopted fish farms were grouped into different clusters according to the results of structure analysis and DAPC. The first and second methods were performed using Arlequin v 3.5 (Excoffier & Lischer, 2010). The first method was based on the hierarchical island model (H) (Excoffier et al., 2009), which can reduce the number of false‐positive outliers. The significance of outliers was assessed using 10,000 simulations, 100 demes, and 5 groups. The second method was the simulation of the finite island model (nH) using the same specifications as the hierarchical island method (H). For both methods, loci presenting *p*‐values ≤0.01 were considered as the putative outliers. The third method was performed using Bayescan v. 2.1 (Foll & Gaggiotti, 2008), with 20 pilot runs, 100,000 iterations, and 50,000 burn‐in steps. The program calculates the posterior odds (PO) based on the probability of a particular locus being under selection using a value of prior odds equivalent to 100:1 and the proportion of loci with a strong increase in *F*ST relative to the other loci. Loci presenting q‐values ≤0.05 were considered the putative outliers. All resulting SNPs were considered in this analysis. To evaluate the effect of SNP outliers on the differentiation of fish farms, a second cluster analysis was performed using IBS and DAPC considering only the putative SNP outliers.

The ddRAD‐tags containing the putative outliers were annotated by BLASTN search against the tambaqui genome in GenBank (GCA_904425465.1). The significance threshold for homologies (e‐value) was <e^−4^. The search for genes was performed when the loci were anchored in the same region within a range of <250 kb on both sides. The genetic variant annotation for each candidate SNP to be under selection was performed using SnpEff (Cingolani et al., 2012).

## 3 RESULTS

### 3.1 SNP identification

Approximately 300 Gb (billions of nucleotide bases) reads were sequenced through the libraries, resulting in an average of approximately 220 M reads per library. After the application of the quality filters on sequences, approximately 180 M reads per library were retained for SNP characterization. On average, approximately 4 M reads per individual were considered, and a total of 207 M reads were excluded due to ambiguous barcodes. For genomic analysis, 199 samples were considered (after individual missingness filtering, mind = 0.4). A total of 6323 SNPs were obtained after aligning the sequences against the tambaqui reference genome (GCA_904425465.1), excluding SNPs with a quality score of <20, minimum allele count of <4 reads, and HWE *p*‐value of <1 × 10^−6^. Subsequently, 1276 SNPs presenting MAF < 0.05 and proportion of missing genotypes >30% were discarded, leaving 5047 SNPs for downstream analyses. All remaining SNPs attained a minimum call rate of 70% individuals in a population (call rate > 0.70).

### 3.2 Genetic diversity

The descriptive statistics of genetic diversity for each population are presented in Table 1 and are based on 1633 SNPs (3414 SNPs were eliminated after LD pruning).

Overall, there were no large differences in diversity parameters among the fish farms and between the farmed and WILD populations. Most farmed populations from Colombia (COL1 to COL5) and Peru (PER) presented lower N_a_, H_o_, and H_e_ values than the farmed populations from Brazil (BR). The diversity estimates of farmed populations from Colombia and Peru were similar to those of the wild population (WILD). Markers with rare alleles and lower MAF values were more abundant in farmed populations from Colombia (e.g., COL5 and COL4), except COL3, whereas farmed populations from Brazil presented higher MAF frequencies. All inbreeding coefficients (F_IS_) were positive, ranging between 0.060 (BR1) and 0.357 (BR3), and the SNP loci were in Hardy–Weinberg disequilibrium in most populations (except COL5 and WILD) (Table 1). The F_IS_ value over all loci was 0.223, and the effective population size (N_e_) was low and variable among the farmed populations, ranging from 9.8 in BRGEN to 75.6 in COL5. N_e_ could not be estimated for WILD because the confidence interval extremely large (infinite), making the values unreliable. N_e_ estimates for COL5 should be interpreted cautiously because of the small number of individuals analyzed. The N_e_ estimates for inbreeding rate (∆F) per generation ranged from 0.9% in BR6 to 5.1% in BRGEN (Table 2).

**TABLE 2:** Effective population size (Ne) parameter based on linkage disequilibrium, considering 1633 SNPs

Sampling site	*N*	Ne	CI 95%	Jackknife CI	∆F
BR1	14	12.1	11.8–12.2	6.6–26.5	0.041
BR2	19	17.8	17.5–18.1	10.5–37.2	0.028
BR3	20	19.9	19.6–20.4	10.8–55.3	0.025
BR4	11	54.7	53.5–62.1	11.1–Inf.	0.009
BR5	15	15.1	15.1–15.8	8.2–40.5	0.033
BR6	18	26.5	25.8–27.1	13.7–95.2	0.018
BRGEN	15	9.8	9.7–10.0	3.6–28.3	0.051
COL1	19	14.0	13.6–14.0	9.5–21.6	0.036
COL2	14	34.8	32.6–35.6	15.6–640.2	0.014
COL3	16	46.9	45.2–49.0	21.0–Inf.	0.011
COL4	12	17.2	16.7–17.8	7.1–188.1	0.029
COL5	6	75.6	42.6–62.6	7.3–Inf.	0.006
PER	14	25.8	24.7–26.4	10.8–2164.4	0.019
WILD	6	Inf.	Inf.–Inf.	8.3–Inf.	Infinite

Abbreviations: CI, confidence interval 95%; inbreeding coefficient (∆F = 1 / 2Ne);Inf.: infinite; *N*, number of breeders for each fish farm.

### 3.3 Population genetic differentiation and structure

**FIGURE 1:**
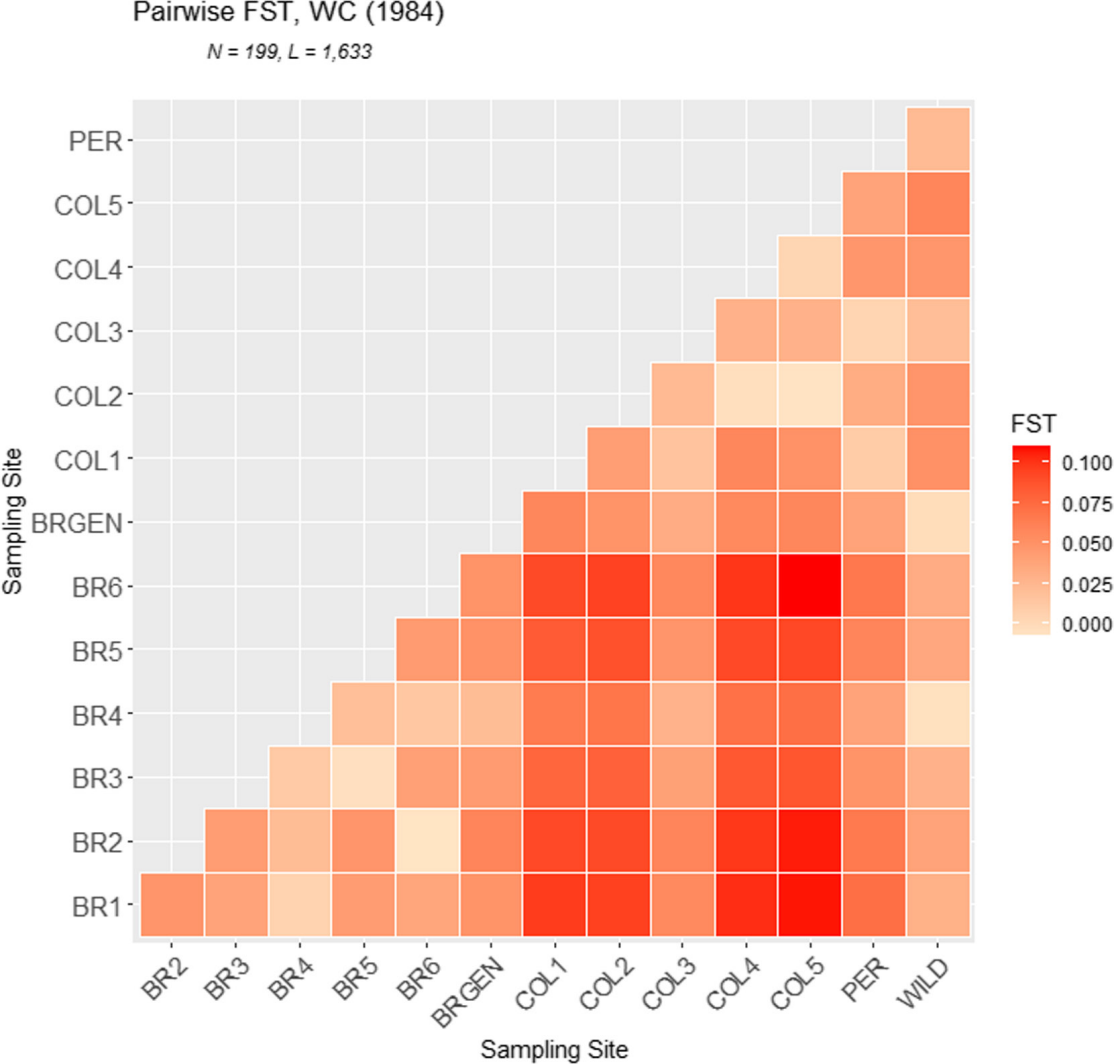
Heatmap showing differentiation between tambaqui stocks from South America based on *F*_ST_

A low but significant genetic differentiation among all populations was detected based on global *F*ST values using SNP loci (*F*_ST_ = 0.064, *p* < 0.05). In *F*ST pairwise analysis (Table 3; Figure 1; Table S1a), low‐to‐moderate genetic differentiation was observed between all population pairs. All values were significantly greater than zero, except for some comparisons involving geographically close populations (shown in bold letters in Table 3); these values ranged from 0.002 for the genetic differentiation between BRGEN and WILD, to 0.109, the highest genetic differentiation detected between BR6 and COL5 (Table 3). The Nei genetic distances showed a similar pattern of little differentiation among farmed populations in Brazil. Meanwhile, the highest genetic distance was noted between the farmed populations in Brazil and Colombia (COL1 to COL5) (Table 3).

**TABLE 3:** Values of population differentiation based on *F*_ST_ (below the diagonal) and Ney genetic distance (above the diagonal)

	BR1	BR2	BR3	BR4	BR5	BR6	BRGEN	COL1	COL2	COL3	COL4	COL5	PER	WILD
BR1	0	0.044	0.037	**0.006**	0.041	0.037	0.042	0.084	0.089	0.047	0.095	0.099	0.059	0.025
BR2	0.048	0	0.035	0.014	0.040	**0.006**	0.046	0.080	0.082	0.046	0.086	0.092	0.058	0.028
BR3	0.039	0.043	0	**0.008**	**0.005**	0.036	0.035	0.067	0.074	0.033	0.076	0.078	0.042	0.015
BR4	**0.005**	0.021	**0.011**	0	0.016	**0.012**	0.014	0.051	0.053	0.018	0.059	0.058	0.030	**0.007**
BR5	0.043	0.048	**0.004**	0.020	0	0.039	0.041	0.070	0.077	0.036	0.080	0.086	0.044	0.024
BR6	0.037	**0.007**	0.041	**0.013**	0.044	0	0.042	0.080	0.083	0.044	0.085	0.092	0.056	0.026
BRGEN	0.049	0.058	0.044	0.020	0.050	0.050	0	0.051	0.044	0.023	0.048	0.054	0.032	**0.007**
COL1	0.097	0.092	0.077	0.065	0.083	0.091	0.057	0	0.040	0.009	0.049	0.051	**0.008**	0.040
COL2	0.095	0.091	0.079	0.067	0.088	0.094	0.048	0.042	0	0.019	**0.007**	**0.006**	0.033	0.046
COL3	0.055	0.058	0.040	0.028	0.048	0.056	0.032	0.016	0.022	0	0.023	0.023	**0.001**	**0.008**
COL4	0.102	0.099	0.084	0.071	0.091	0.098	0.055	0.057	**0.004**	0.028	0	**0.0001**	0.039	0.042
COL5	0.107	0.106	0.086	0.072	0.092	0.109	0.057	0.050	**0.006**	0.029	**0.003**	0	0.036	0.048
PER	0.072	0.065	0.049	0.039	0.058	0.066	0.039	**0.010**	0.032	**0.004**	0.046	0.039	0	**0.009**
WILD	0.029	0.039	**0.029**	**0.004**	0.036	0.033	**0.002**	0.050	0.048	**0.020**	0.047	0.057	**0.022**	0

Note

All values were significant (*p*‐value < 0.05) except those shown in bold letters.

**FIGURE 2:**
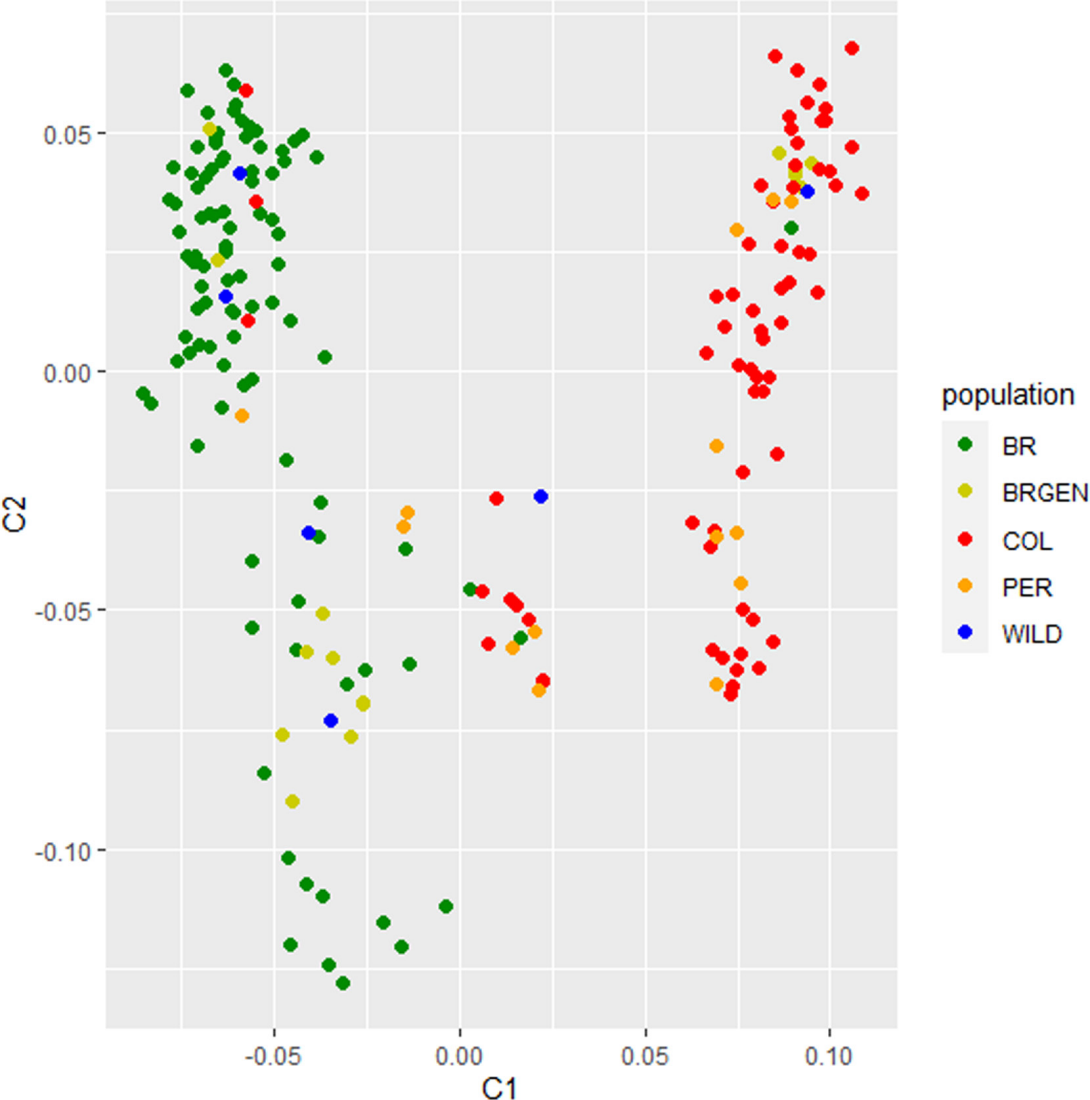
Population genetic analysis of tambaqui stocks from South America. Multidimensional scaling (MDS) analysis resulted from 199 individuals. Individuals were plotted according to their coordinates on the first two components (C1 and C2)

**FIGURE 3:**
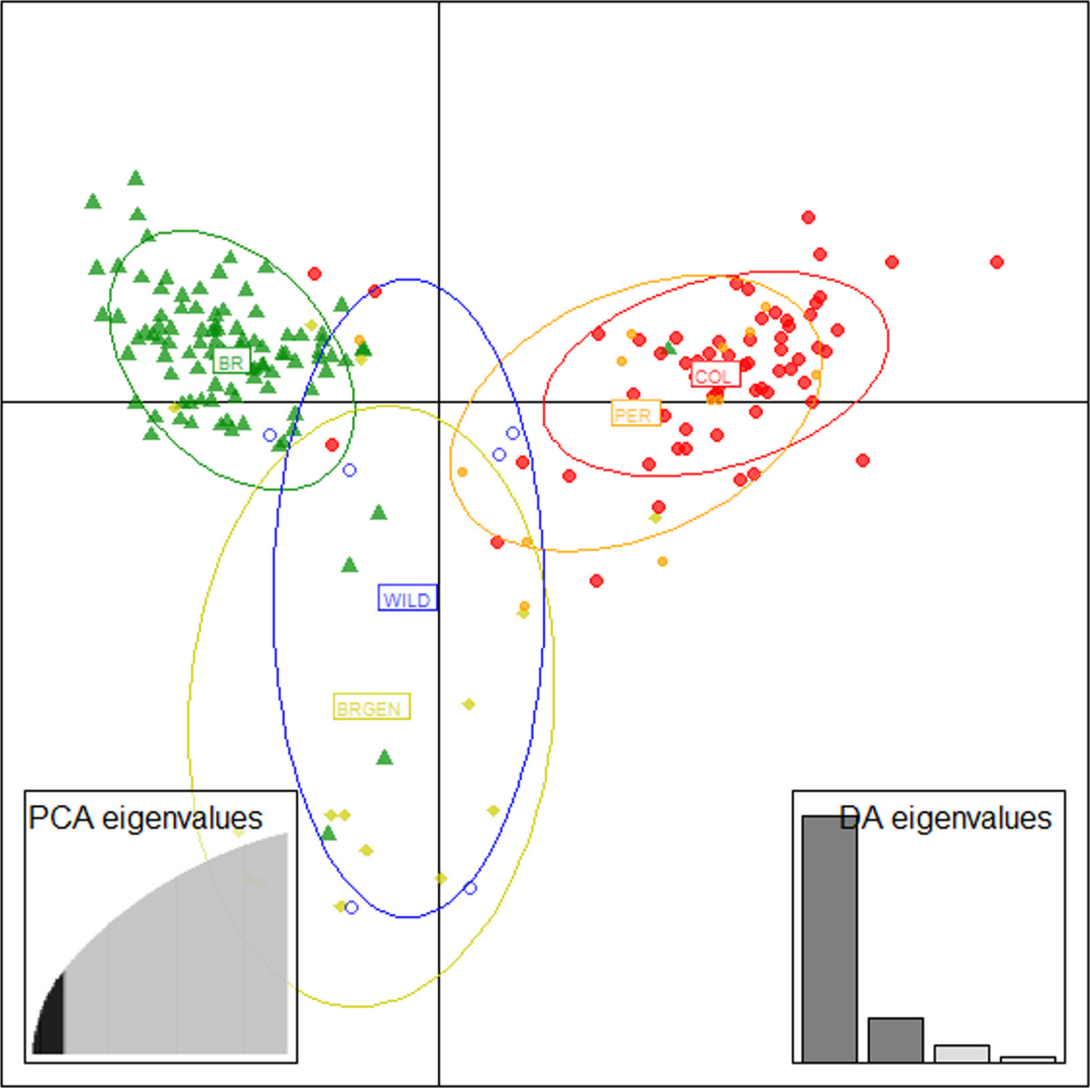
Plot of discriminant analysis of principal components (DAPC) showing relationship of 199 individual fish color coded by sample site, representing the structure between tambaqui stocks from South America. Twenty five PCs were retained using a‐score optimization

The genetic distance between these populations was evaluated based on clusters by calculating the average measures of IBS of the SNP markers and then summarizing them using MDS to determine population structures in different countries (Figure 2). The results were posteriorly confirmed by DAPC, retaining 25 PCs through a‐score optimization (Figure 3). Samples from Brazil and Colombia formed separate clusters, confirming the genetic differentiation between the farmed populations. Furthermore, genetic similarity was observed between farmed populations from Colombia and Peru and between WILD and BRGEN (Figures 2 and 3).

**FIGURE 4:**
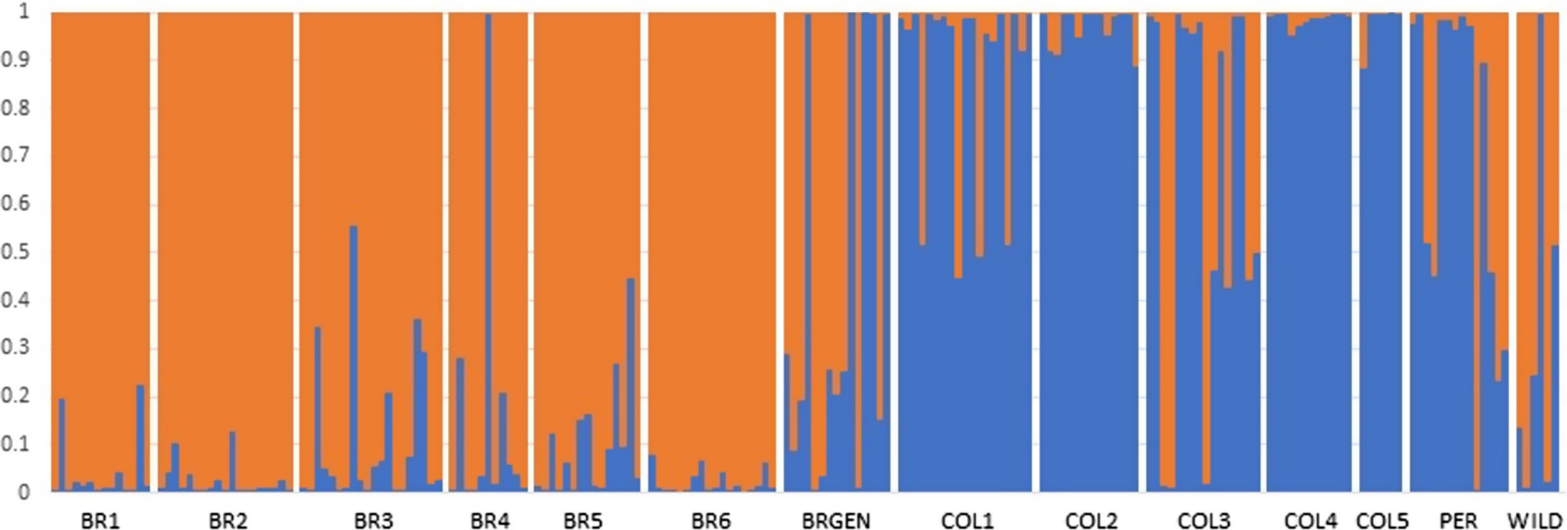
Analysis of genetic structure of tambaqui stocks from South America. Genetic structure was analyzed approaching K = 2 according to Delta K statistics. Each vertical bar represents an individual. Populations are separated by vertical white bars. The color proportions of each bar correspond to the estimated fractions of association of the individuals in each of the clusters

To assess the level of mixing between samples, cluster analyses using the Bayesian model were performed based on the distribution of ΔK (Figure 4). The results indicated that K = 2 was the most adequate value for explaining the population structure of the farmed populations of tambaqui in South America. Overall, all genetic structure analyses identified the presence of two main clusters: (1) BR1, BR2, BR3, BR4, BR5, and BR6 (orange group) and (2) COL1, COL2, COL4, and COL5 (blue group). COL3, PER, WILD, and BRGEN populations were considered a mixture of both genetic clusters, although the cluster found in their respective geographical regions was the most abundant (i.e., cluster 1 for WILD and BRGEN and cluster 2 for COL3 and PER) (Figure 4).

Based on the above population clusters, two AMOVAs were performed according to the structure hypothesis (Model I: group 1: BR1, BR2, BR3, BR4, BR5, BR6, and BRGEN and group 2: WILD, COL1, COL2, COL3, COL4, COL5, and PER) and DAPC results (Model II: group 1: BR1, BR2, BR3, BR4, BR5, and BR6; group 2: BRGEN and WILD; and group 3: COL1, COL2, COL3, COL4, COL5, and PER). There were no significant differences between the two models. The highest proportion of genetic variance was present within the populations (*F*_ST_) (~93%), whereas the genetic variance among groups (*F*_CT_ = ~3%) and among populations within groups (*F*_SC_ = ~3%) was significantly low (Table 4).

**TABLE 4:** Analysis of molecular variance (AMOVA) of tambaqui stocks *Colossoma macropomum*

	*F*‐statistic	Variance component	% Variation
Model I—Structure (K = 2)			
Among groups (*F*_CT_)	0.033*	10.33	3.31
Among populations within groups (*F*_SC_)	0.035*	10.60	3.40
Within populations (*F*_ST_)	0.067*	290.96	93.29
Model II—DAPC analysis			
Among groups (*F*_CT_)	0.035*	10.93	3.51
Among populations within groups (*F*_SC_)	0.030*	9.14	2.94
Within populations (*F*_ST_)	0.064*	290.96	93.55

Note

For Model I, the populations were grouped following population units (K) described by structure analyses (K = 2), while for model II, the populations were grouped by DAPC results. *p‐value > 0.01.

### 3.4 Regions with SNP outliers and their putative genes

The outliers were detected using two groups based on the results of structure and DAPC analyses; the first group was composed of farmed populations from Brazil (BR1, BR2, BR3, BR4, BR5, and BR6), and the second group was composed of farmed populations from Colombia (COL1, COL2, COL3, COL4, and COL5) and Peru (PER). The analysis was performed using two different software and three different methods. A graphical representation of the outliers identified by the Arlequin (nH, H) and Bayescan methods is provided in Figure S1. A total of 82 SNPs were identified as outliers, considering all three approximations. Of these, two outliers (2.4%) were detected by all methods, 12 outliers (14.6%) by the Arlequin methods (nH, H), and only 1 (1.2%) identified by the Bayescan method alone. The corresponding outliers were characterized and associated with functional genes, as shown in Table 5. Most outliers were identified exclusively by the hierarchical island model (nH) (67; 81.8%) (Figure S1) and were characterized and associated with functional genes (see Table S1b). The Bayescan analysis revealed moderate evidence of selection considering the three detected outliers [log10 (PO) = 1.000–1.833; *q*‐value < 0.05]. The Arlequin analysis revealed outliers with apparent evidence of selection, presenting high observed *F*ST values for both the nH (obs *F*_ST_ = 0.227–0.411) and H (obs *F*_ST_ = 0.335–0.430) (*p*‐value < 0.01) models. The visualization of IBS and DAPC (5 PCs retained) revealed two distinct clusters: one formed by the COL and PER samples and the other formed by the BR samples (Figure S2a and b).

**TABLE 5:** Summary of outliers identified by the three utilized methods

SNP ID	BayeScan			Arlequin (H)		Arlequin (nH)		Blastn	
	Log10(PO)	*q*‐value	*F*_ST_	Obs *F*_ST_	*p*‐value	Obs *F*_ST_	*p*‐value	Gene	Annotations
cmj_57835:205	1.833	0.014	0.255	0.361	0.008	0.291	0.005	*ATF6*	I,ST
cmj_49626:177	1.000	0.052	0.211	0.380	0.011	0.301	0.002	*ENO1A*	ST
cmj_139241:10	1.269	0.033	0.240	–	–	–	–	*IRF4*	I
cmj_40107:214	–	–	–	0.413	0.001	0.335	0.001	*OPN3*	ST
cmj_115873:85	–	–	–	0.430	0.003	0.354	0.002	*SBK1*	ST
cmj_13492:21	–	–	–	0.427	0.005	0.301	0.002	*ULK1*	ST
cmj_158265:204				0.395	0.006	0.347	0.0003	*PLXNA2*	ST
cmj_3210:95				0.371	0.006	0.296	0.004	*WRNA*	S
cmj_185105:197	–	–	–	0.397	0.006	0.304	0.004	*INO80C*	ST
cmj_47461:171				0.383	0.007	0.312	0.0007	*SPRED1*	I
cmj_34560:96	–	–	–	0.361	0.007	0.283	0.006	*RBP2B*	S
cmj_44265:124	–	–	–	0.389	0.008	0.311	0.004	*ERCC3*	I, ST
cmj_97663:203	–	–	–	0.391	0.008	0.311	0.004	*FGF4*	I
cmj_85247:50				0.352	0.009	0.280	0.008	*NMS*	ST
cmj_919:262	–	–	–	0.335	0.010	0.281	0.007	*ZFAND5A*	I

Abbreviations: H, hierarchical island model. Blastn annotations: I, genes related to defense response against pathogens; nH, finite model; S, genes related to sensory system; ST, genes related to stress responses.

Regarding annotation of the putative SNP outliers, 36.6% SNPs were found in transcript regions, 35.1% were classified as intron variants, 9.3% were classified as upstream gene variants, and 9.0% were located in intergenic regions, and the closest gene was identified. Regarding the effects of SNP outliers, we imputed the overall impact of all variants as largely modifying (97.6%) (see Table S1c). Mining of the ddRAD‐tags containing the outliers found genes with important functions surrounding these markers (Table 5; Table S1B). Among the 14 outliers detected by different methods and identified exclusively by the Bayescan method (Table 5), nine outliers (60.0%) were surrounded by genes related to stress response (ST) and six outliers (40.0%) were related to defense response against pathogens under stressful conditions (I) (Table 5).

## 4 DISCUSSION

### 4.1 Levels of genetic diversity and population structure

The present study used an SNP dataset (1633 SNPs) to unveil the genetic variability and population structure of farmed tambaqui populations in South America. Considering the massive amount of genetic data, the genetic diversity (H_e_ and MAF values) in the farmed populations was moderate or high, contrary to the pattern of low genetic variability in farmed tambaqui populations demonstrated in a previous analysis based on mitochondrial DNA and microsatellite data (Aguiar et al., 2018; Ferreira et al., 2019; Gonçalves et al., 2018). Diversity estimates were higher for the farmed populations from Brazil than for the farmed populations from Colombia and Peru. The F_IS_ values were positive and high for all populations, and the loci were in Hardy–Weinberg disequilibrium in most populations, revealing homozygous excess. This pattern can be explained by common breeding practices in aquaculture, in which random mating is typically not performed or by broodstock exchange between farmed populations without proper management, which may increase the proportion of homozygous genotypes for different alleles that might have been originally fixed in the farm of origin, due to inbreeding or genetic drift. Moreover, the lack of control of homozygous excess was reflected in the low N_e_ estimates for most farmed populations, being lower than a minimum of 50 animals per generation to control inbreeding, as recommended by the Food and Agriculture Organization of the United Nations (FAO, 1998).

Although the genetic diversity in farmed populations from Brazil may be favored due to the exchange of stocks among breeders, broodstocks in all analyzed populations were not appropriately renewed by individuals originating, for instance, from future progenies or wild environments. Furthermore, the reduced number of breeders allied to the lack of directed mating of nonparental individuals can decrease genetic diversity over the generations of farmed populations from South America (Brown et al., 2005; Mastrochirico‐Filho et al., 2019).

The loss of genetic variability in farmed tambaqui has been described in Brazilian hatcheries, suggesting the lack of genetic monitoring and adequate breeding management in farmed populations of the species (Aguiar et al., 2018; Ferreira et al., 2019; Gonçalves et al., 2018). Therefore, our results can be attributed to the combination of a limited number of founders in farmed populations without proper genetic management, leading to random genetic drift and inbreeding caused by domestication processes, which may have compromised the genetic diversity of the farmed populations. To minimize the impacts of the low number of individuals used in the hatcheries, additional studies on the genomics and management of South American stocks are fundamental for the proper functioning of initial breeding programs.

Analysis of genetic differentiation between the farmed populations based on pairwise *F*ST values and Nei distances revealed higher genetic differentiation of Brazilian stocks from Colombian and Peruvian stocks. Meanwhile, genetic similarity was observed between farmed populations from Colombia and Peru as well as between WILD and BRGEN (Figures 2 and 3). However, the observed genetic differentiation values were not expressive, highlighted by the overall *F*ST estimate of 0.064 (*p*‐value < 0.05). Moreover, AMOVA revealed the highest significant differentiation within populations (93.29%, *p* < 0.01) and not between groups (3.31%, *p*‐value < 0.01). Considering the panmictic nature of the wild populations from the Amazon Basin (Santos et al., 2007), the low‐to‐moderate genetic differentiation between farmed populations from the northern and central regions of South America was significant, exhibiting unique genetic structures. Primarily, the genetic differences observed between the studied farmed populations, and confirmed by structure and DAPC analyses, may be related to the geographical isolation of tambaqui production from the central region of Brazil, where the access to farmed populations from northern region is limited, restricting the gene flow due to the geographical barrier and the exchange of broodstocks among the hatcheries. Simultaneously, stock foundation from captured wild individuals did not likely favor the high genetic differentiation between fish farms, as evidenced by the low of *F*ST values between the WILD and farmed populations. Consistently, the panmictic nature of tambaqui populations in the Amazon River has been documented (Santos et al., 2007). Interestingly, farmed populations from Colombia and Peru, despite being located the nearest to the WILD populations, showed a higher genetic differentiation from the WILD populations, emphasizing the lack of renewal of broodstocks by new individuals originating from the wild and poor management practices.

### 4.2 SNP outliers and their putative genes

The application of large‐scale SNP datasets has become increasingly popular for the detection of selection signatures due to increased marker density and, consequently, increased genomic coverage (Gutierrez et al., 2016). In the present study, 82 SNPs (1.62%) were identified as outliers using a large SNP dataset (~5 K). Previous studies analyzing selection signatures in aquaculture species have detected few regions potentially under selection. For instance, Gutierrez et al. (2016) identified moderate evidence of 44 putative selection signatures in an Atlantic salmon population using a 6.5K SNP array. Furthermore, Mäkinen et al. (2014) analyzed three populations of Atlantic salmon and found little evidence of signatures of selection using the Bayescan software.

In the present study, although significant evidence of selection was detected in 82 genomic regions, only three outliers were detected by the Bayescan method, which is considered a more conservative approach than the Arlequin methods, and only two outliers were detected by all selected methods. Although previous studies have reported a high rate of false‐positive outliers using the Arlequin and Bayescan methods (Narum & Hess, 2011), both approaches have been used to detect selection signatures in aquaculture species (Gutierrez et al., 2016; Liu et al., 2015; Vera et al., 2019). Given the differences between the methods used to identify the evidence of selection and caution with which these loci under selection should be evaluated due to the high false‐positive rate, we considered the most consistent outliers supported by both Arlequin methods, in addition to those identified exclusively by the Bayescan method. The detected outliers may be related to domestication processes in many parts of South America. Tambaqui production has become more intensive over the years and involves diverse farm management practices, including handling, confinement, fertilization, and other operations. Nevertheless, intensive management procedures may produce some level of disturbance, leading to stress responses and, consequently, poor fish performance. Additionally, tambaqui is considered a susceptible species to extreme environmental conditions, and although its production is possible in a wide range of South American territories, fish farmers are discouraged from cultivating this species during the winter, specifically in the southern and southeastern regions (subtropical area of Brazil), when there are long periods of low temperature (Fernandes et al., 2018). Temperature fluctuations affect the physiological processes of all fish, particularly under stressful conditions (Alfonso et al., 2020). These changes in the physiological processes may increase susceptibility to diseases, such as those caused by parasites and bacteria, as already described in cases of disease outbreaks during tambaqui production (Ariede et al., 2020; Lira et al., 2020).

Of the three outliers detected by the Bayescan method (q‐value < 0.05) and the 12 outliers detected by both Arlequin methods (*p*‐value < 0.01), nine were related to stress response and six were related to immunity (Table 5). However, we were particularly interested in the six SNPs that were identified as outliers in this analysis (cmj_57835:205, cmj_49626:177, cmj_139241:10, cmj_115873:85, cmj_13492:21, cmj_158265:204, and cmj_85247:50). These outliers were found to be associated with genes that integrate the genetic components of stress responses to body temperature, confinement, and management practices, such as handling and feeding deprivation. The loci harboring the outliers cmj_57835:205 and cmj_158265:204 have been implicated in thermal stress response, and they were, respectively, associated with cyclic AMP‐dependent transcription factor ATF‐6 alpha (*ATF6*) (Wang et al., 2016) and plexin A2 (*PLXNA2*), which were involved in the muscle development and growth of threespine stickleback (*Gasterosteus aculeatus*) under different temperatures (Metzger & Schulte, 2018); Outliers cmj_49626:177 and cmj_115873:85 showed homology with proteins involved in stress response to handling and confinement. Meanwhile, cmj_49626:177 was associated to enolase 1a (*ENO1a*), which is involved in stress response to repeated handling in Senegalese sole; suboptimal rearing conditions in Gilthead seabream (Raposo de Magalhães et al., 2021); and hypoxia/thermal stresses in zebrafish larvae (Long et al., 2015), pikeperch (Swirplies et al., 2019), and tambaqui (Prado‐Lima & Val, 2016). Moreover, cmj_115873:85 was associated with a serine/threonine protein kinase (*SBK1*), which has been mapped to differentially expressed transcripts related to handling and confinement stress response in rainbow trout (Gonzalez‐Pena et al., 2016). Stress response to food deprivation has been widely discussed in fish farming procedures aimed at evaluating the metabolic regulation and reallocation of energy under these critical periods (Lutfi et al., 2018). Two outliers (cmj_13492:21 and cmj_85247:50) were surrounded by genes encoding the serine/threonine protein kinase *ULK1*, which is involved in autophagy in response to starvation and nutrition deficiency in farmed species (Fan et al., 2019; Wu et al., 2020), and neuromedin S (*NMS*), which is involved in the neuroendocrine regulation of feeding and, possibly, in the reallocation of energy under stress response (Li et al., 2015).

In tambaqui production, domestication is a very recent process, and it has not yet been fully implemented, hampering the presence of a large number of fixed outliers over the generations. The lack of genetic monitoring and inadequate breeding management may compel fish farmers to repeatedly acquire new broodstock from the wild environment, interrupting the fixation of selection signatures and loci involved in adaption to local conditions. Nevertheless, the present study is the first to aim at identifying the loci under selection in tambaqui. Although these findings must be carefully evaluated, they will serve as a basis for expanding our understanding of the genetics of adaptability of fish to farmed systems.

To the best of our knowledge, the present study is the first to investigate the genetic composition of tambaqui stocks from the major producers in South America using genome‐wide SNP markers. Therefore, our results provide fundamental knowledge of the genetic profiles of tambaqui stocks from different parts of South America, considering the importance of generating subsidies for the progress of its production. The genetic diversity and structure of stocks highlight the risk of inbreeding and emphasize the necessity of directed mating of stocks to maintain genetic variability and avoid inbreeding. Evidence of selection signatures in tambaqui stocks associated with different farming environments was detected. Even though the number of outliers was low, most were associated with stress tolerance and immunity. Therefore, further studies are warranted to validate these predicted genes as important candidates to improve tambaqui production in many parts of South America.

## CONFLICT OF INTEREST

The authors declare no conflict of interest.

## Supporting information

Fig S1. Venn diagram showing the representation of the outliers identified by Arlequin (finite model: nH, hierarchical model: H) and Bayescan methods in the tambaqui populations.

Fig S2a. Population structure analyses based on the putative SNP outliers using IBS method.

Fig S2b. Population structure analyses based on the putative SNP outliers using DAPC method, visualized after retaining 5 optimum PCs.

Table S1

## Data Availability

The raw fastq files obtained by ddRAD‐Seq methods for SNP discovery in tambaqui are available in the National Center for Biotechnology Information (NCBI) Sequence Read Archive (SRA) (PRJNA680381). The data that support the results of this study (list of SNPs and SNPs under selection) are openly available in the DRYAD Digital Repository at https://doi.org/10.5061/dryad.mgqnk991h.
