# Supplementary figures and images for: Genomic selection signatures in farmed Colossoma macropomum from tropical and subtropical regions in South America
Source: Evol Appl. 2022 Feb 24;15(4):679–93. doi: 10.1111/eva.13351 (PMC9046916; doi:10.1111/eva.13351)

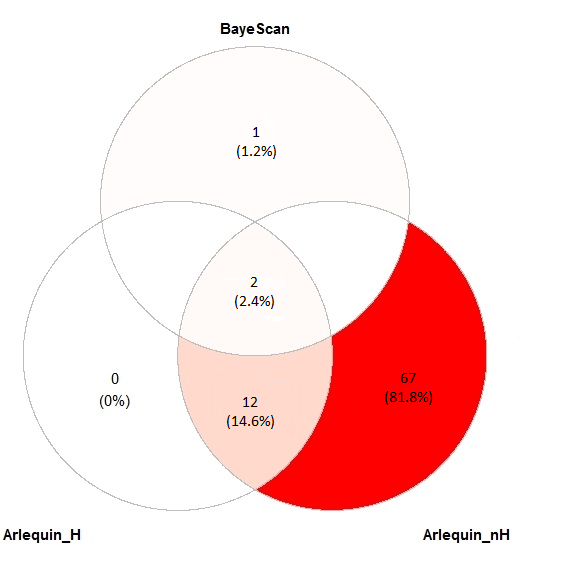

Supplement: Supplementary file 1 — Fig S1. Venn diagram showing the representation of the outliers identified by Arlequin (finite model: nH, hierarchical model: H) and Bayescan methods in the tambaqui populations. [file EVA-15-679-s002.tiff]

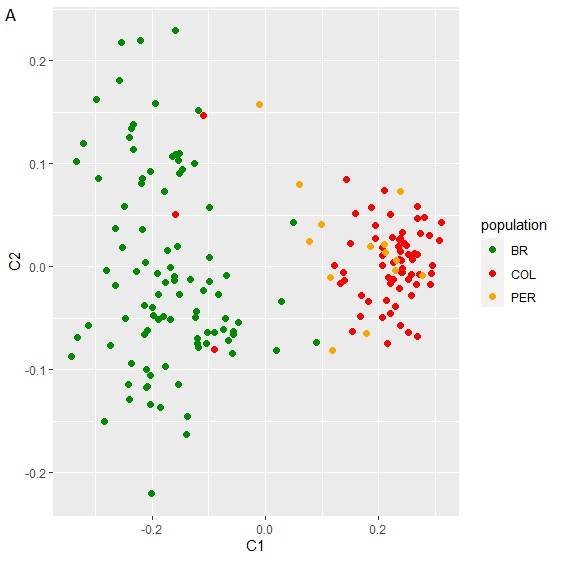

Supplement: Supplementary file 2 — Fig S2a. Population structure analyses based on the putative SNP outliers using IBS method. [file EVA-15-679-s003.tiff]

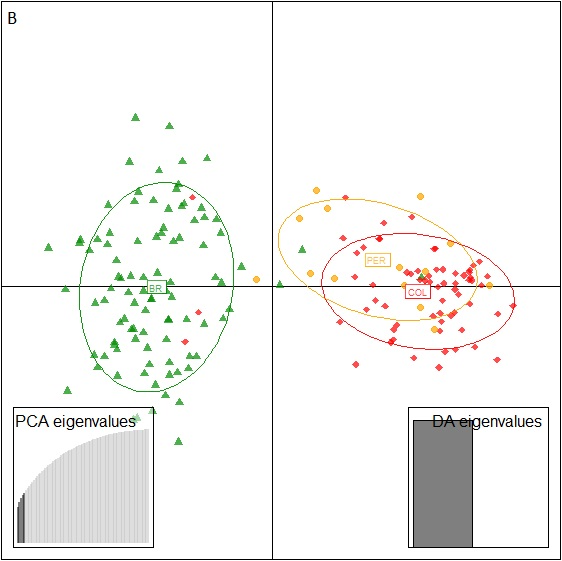

Supplement: Supplementary file 3 — Fig S2b. Population structure analyses based on the putative SNP outliers using DAPC method, visualized after retaining 5 optimum PCs. [file EVA-15-679-s004.tiff]
